# Determining structural ensembles of flexible multi-domain proteins using small-angle X-ray scattering and molecular dynamics simulations

**DOI:** 10.1007/s13238-015-0162-4

**Published:** 2015-05-06

**Authors:** Yonghui Zhang, Bin Wen, Junhui Peng, Xiaobing Zuo, Qingguo Gong, Zhiyong Zhang

**Affiliations:** Hefei National Laboratory for Physical Science at Microscale and School of Life Sciences, University of Science and Technology of China, Hefei, 230026 China; Advanced Photon Source, Argonne National Laboratory, Chicago, IL 60437 USA

**Dear Editor,**

A multi-domain protein consists of two or more well-folded domains connected by flexible linkers, which may lead to large scale inter-domain motions related to the protein function. Therefore, it is appropriate to represent a flexible multi-domain protein by an ensemble of structures containing multiple conformational states (Bernadó and Blackledge, 2010). Although X-ray crystallography is currently the most popular technique for structure determination, it would be rather challenging to solve structure of such a flexible multi-domain protein since it is hard to obtain high-quality crystals if the protein does not stabilize in a dominant conformation. Solution nuclear magnetic resonance (NMR) is able to investigate structure and dynamics of flexible multi-domain proteins with modest sizes, but it may not be easy to obtain enough long-range NMR restraints in order to determine orientations between the domains. Small-angle X-ray scattering (SAXS) has made substantial progress over the past decades (Graewert and Svergun, 2013), which can provide valuable structural information, such as the sizes and shapes, of proteins. SAXS is particularly useful in characterizing the flexibility of a multi-domain protein because the scattering profile contains the information of multiple conformations of the protein and their relative population in solution. Although the resolution of SAXS is inherently low since the complex protein structure is reduced to a one-dimensional orientationally averaged profile, it can serve as a complementary experiment to these high-resolution techniques. For example, structures of individual domains can be solved by NMR, and SAXS provides a restraint between the domains (Grishaev et al., 2005).

Computer simulations play important roles in combining the high and low-resolution structural data to investigate flexible multi-domain proteins (Bernadó and Blackledge, 2010; Yang, 2014). In such applications, many groups have used a screening-after-sampling strategy (Bernadó et al., 2007; Pelikan et al., 2009; Różycki et al., 2011; Schneidman-Duhovny et al., 2012; Wen et al., 2014; Yang et al., 2010), that is, a structure pool of the protein with a large number of different conformations is generated by simulations beforehand, and then an ensemble of structures is screened out of the pool to best reproduce the SAXS profile. Among various computational methods, molecular dynamics (MD) simulation is widely used in studying protein dynamics (Dror et al., 2012). With the increasing computer power and development of advanced algorithms, nowadays MD simulations can reach a time scale of microsecond (Arkhipov et al., 2013) to millisecond (Ma and Schulten, 2015). In this work, we show the potential of combining experimental SAXS data and extensive MD simulations to determine structural ensembles of multi-domain proteins.

The formin binding protein 21 (FBP21) is a structural component of the mammalian spliceosomal A/B complex and has functionality in pre-mRNA splicing (Bedford et al., 1998). NMR structure of its tandem WW domains (denoted as FBP21-WWs) has been solved that consists of 75 amino acid residues (Huang et al., 2009). The two WW domains, called WW1 (residues 6–32) and WW2 (residues 47–73), respectively, are connected by a flexible linker, which leads to high mobility between them and enables their cooperative interactions with different ligands. By observing 20 structural models in the NMR ensemble (PDB entry 2JXW), the internal structures of both WW1 and WW2 are converged, but their relative orientations are various (Fig. 1A). The ^15^N relaxation experiments also suggest that there exists significant inter-domain dynamics in FBP21-WWs. Therefore, it is important to explore relative domain orientations in the protein, which is however un-determined due to the lack of NMR restraints between the domains.

**Figure 1:**
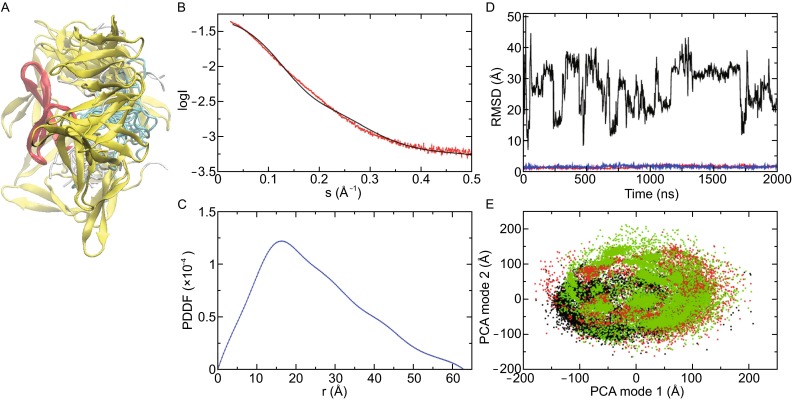
**The NMR ensemble, SAXS data, and MD simulations of FBP21-WWs**. (A) The NMR ensemble containing 20 structural models, which are superimposed by the WW1 domain (colored in red) in order to show different orientations of the WW2 domain (colored in yellow). (B) The experimental SAXS profile of FBP21-WWs (red solid line), and the calculated SAXS curve of the NMR ensemble (black solid line). The discrepancy χ between them is 0.63. (C) The PDDF curve. (D) Domain motions in the single-2µs MD simulation measured by RMSD. The RMSD curve of WW1, WW2 and the two WW domains is colored in red, blue and black, respectively. For each domain, only its Cα atoms were used to compute RMSD. For the two WW domains all the configurations in the trajectory were superimposed to the starting structure by Cα atoms in WW1, while RMSD were calculated using Cα atoms in both domains. (E) Projection of the different trajectories onto the 2D subspace defined by the first two PCA modes with the largest eigenvalues. Projection of single-2µs, comb1-2µs and comb2-2µs is colored in black, red and green, respectively.

We collected SAXS data of FBP21-WWs. The one-dimensional SAXS profile (red curve in Fig. 1B) shows a high signal-noise ratio with *q* up to 0.5 Å^−1^. The peak of pair distances distribution function (PDDF) is located at around 16.3 Å, but the maximal distance *D*_*max*_ can be longer than 62.0 Å (Fig. 1C). This tailing shape of PDDF suggests that the average conformation of FBP21-WWs in solution is not globular but rather extended. The radius of gyration (*R*_g_) of the protein estimated from Guinier analysis is about 19.0 Å. For each structural model in the NMR ensemble, we back-calculated its theoretical SAXS curve and get the average values (Eqn. S2). The SAXS profile of the NMR ensemble (black curve in Fig. 1B) does not fit the experimental curve well, with the discrepancy χ (Eqn. S1) equals to 0.63. *R*_*g*_ of the 20 NMR models, ranging from 14.6 to 18.3 Å, are not consistent with the value given by SAXS. Therefore, we need to optimize structural ensembles of FBP21-WWs, in order to better reproduce the experimental SAXS data.

To explore different conformations of FBP21-WWs, multiple MD simulations were performed (Table S1). Starting from model 1 in the NMR ensemble, a 2 μs MD simulation was carried out (denoted as single-2µs). From each of the 20 models, two 100 ns MD simulations were run independently. These short simulations were then combined into two 2-μs trajectories (denoted as comb1-2µs and comb2-2µs, respectively). Intra- and inter-domain motions were firstly estimated by computing root mean square deviations (RMSD) of different MD trajectories with respect to their starting structures. The RMSD of the two domains (red and blue curves in Fig. 1D) are both small, with the average value 1.5 ± 0.4 and 1.6 ± 0.3 Å, for WW1 and WW2, respectively. The results indicate that the internal structures of the two WW domains are converged, which are in agreement with the NMR results. RMSD of the two WW domains was calculated after superimposing all the conformations by WW1, in order to reflect inter-domain motions in the simulations. The absolute values and fluctuations of inter-domain RMSD (black curve in Fig. 1D) are dramatically larger than those within each domain, which suggest that the linker region is very mobile and the two WW domains can indeed take various orientations. Time evolution of the angle between the domains also supports the above notion (Fig. S1). We then combined all the three trajectories of FBP21-WWs to perform principal component analysis (PCA). The results indicate that the first two PCA modes with the largest eigenvalues contribute about 66% of the total fluctuation in the protein. The three trajectories were projected onto the two-dimensional subspace defined by these two PCA modes (Fig. 1E). It looks like the conformations in comb1-2µs (red) and comb2-2µs (green) not only cover the most of conformational space sampled by single-2µs (black), but also reach a large portion of other regions in the essential subspace. That is to say, multiple short simulations starting from different conformations can sample a broader region of the conformational space than a single long simulation.

Ensemble optimization method (EOM) (Bernadó et al., 2007) was used for selecting ensembles from the three trajectories, respectively, to fit the SAXS data. Fig. 2A shows an ensemble from single-2µs. Their *R*_*g*_ are from 16.5 to 22.0 Å, and the average value is 18.3 Å. The theoretical SAXS profile agrees with the experimental curve fairly well with χ = 0.24 (Fig. 2B). In an ensemble chosen from comb1-2µs (Fig. 2C), the *R*_*g*_ values are from 15.1 to 22.9 Å with the average 19.2 Å. The χ value of this ensemble in fitting the experimental SAXS profile is only 0.17 (Fig. 2D), which is better than that from single-2µs (Fig. 2B). The ensemble from comb2-2µs (Fig. 2E) contains conformations with *R*_*g*_ ranging from 15.4 to 23.6 Å (the average is 19.0 Å), which has a χ of 0.18 when fitting the experimental data (Fig. 2F). In the three ensembles selected by EOM, the majority of conformations show similar domain orientations. However, in the ensembles from comb1-2µs (Fig. 2C) and comb2-2µs (Fig. 2E), there are several additional conformations with different domain orientations that do not exist in the ensemble from single-2µs (Fig. 2A). Since the two combined trajectories have better sampling in the conformational space of FBP21-WWs than the single MD, the former two are superior in reproducing the experimental SAXS profile than the latter. Those conformations in the comb1-2µs (Fig. 2C) and comb2-2µs (Fig. 2E) ensembles look fairly similar in domain orientations, which may suggest that the results are reliable since they are from independent simulations. In the NMR ensemble (Fig. 1A), domain orientations of the conformations are quite diverse because there are not any restraints between the domains when refining the structures. By applying the SAXS restraint, the protein seems to prefer some conformational states with certain domain orientations although the linker is very mobile. According to ^15^N relaxation experiments, the two WW domains have similar *R*_2_/*R*_1_ ratio, suggesting that there remains some limitation to the flexibility between them (Huang et al., 2009). Our results support this notion, which can be further validated by more NMR experiments, such as residual dipolar coupling (RDC), paramagnetic relaxation enhancement (PRE) or pseudo-contact shift (PCS). These conformations in the SAXS-optimized ensembles can be roughly classified into two states, one includes compact conformations with *R*_*g*_ smaller than 18 Å, and the other represents extended conformations of the protein with *R*_*g*_ larger than 20 Å.

**Figure 2:**
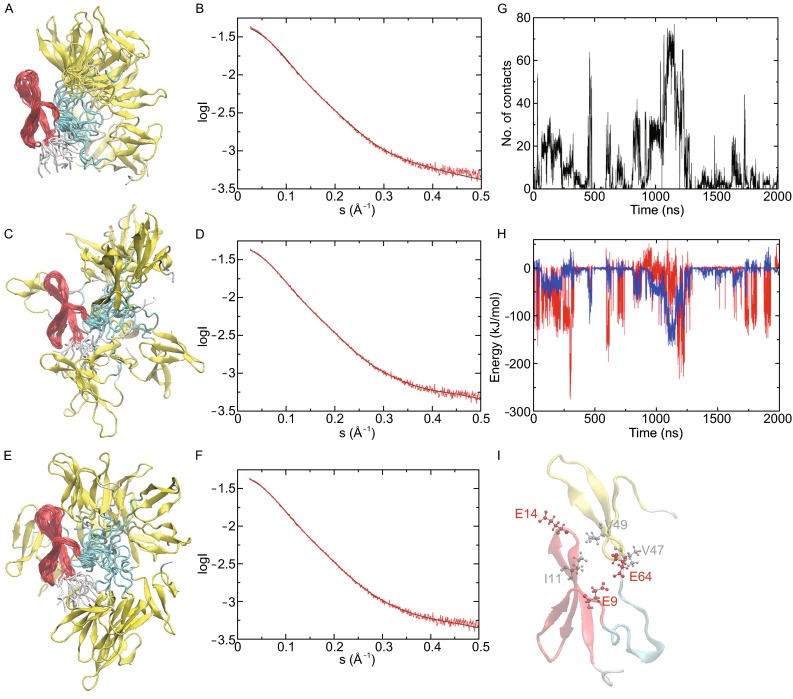
**Ensembles of FBP21-WWs optimized by SAXS, and interaction analysis between the domains**. (A) The EOM ensemble from single-2µs. (B) The calculated SAXS curve (black solid line) of the EOM ensemble from single-2µs with χ = 0.24 compared to the experimental SAXS curve (red solid line). (C) The EOM ensemble from comb1-2µs. (D) The calculated SAXS curve of the EOM ensemble from comb1-2µs with χ = 0.17 compared to the experimental SAXS curve. (E) The EOM ensemble from comb2-2µs. (F) The calculated SAXS curve of the EOM ensemble from comb2-2µs with χ = 0.18 compared to the experimental SAXS curve. The structures in (A), (C) and (E) are superimposed and colored as those in Fig. 1A. (G) Number of contacts between the two WW domains in single-2µs. (H) Van der Waals (blue solid line) and electrostatic (red solid line) interactions between the domains in single-2µs. (I) A representative conformation showing some contacts between the domains, in which hydrophobic residues are colored in gray and charged residues are colored in red.

Atomistic MD simulations can not only sample different conformations of the protein, but also be used to interpret why FBP21-WWs may take both compact and extended conformations in solution. We have analyzed inter-domain interactions in details, and Fig. 2G shows the number of contacts between the two WW domains in single-2µs. For two residues from the different domains, if the distance between any of their heavy atoms is within 6.0 Å, we define a contact between the two residues. There is a large fluctuation of the contacts during the simulation, which suggest that the two WW domains can be close to each other with more than 70 contacts, or far apart without any interaction. It has been found that only 15 out of all the contacts can exist longer than 10% but shorter than 20% of the simulation time. Interaction energies between the two domains were computed (Fig. 2H). It is clear that van der Waals energies (blue curve in Fig. 2H) are highly correlated with the number of contacts (Fig. 2G), that is to say, the more contacts, the more favorable van der Waals interactions are between the domains. However, electrostatic energies (red curve in Fig. 2H), although fluctuate largely, are generally negatively-correlated with the van der Waals energies. A representative conformation of FBP21-WWs is shown in Fig. 2I. When the two domains are close, their favorable interactions are mainly from some hydrophobic residues, such as Ile11-Val49. In the meantime, there exist a number of unfavorable contacts between charged residues like Glu9-Glu64. Our results may explain why none of the inter-domain contacts is stable enough during the simulation. Those competing interactions (van der Waals vs. electrostatic) between the two WW domains, together with the mobile linker, make the protein able to transit between the compact and extended conformations in solution, as that described by the SAXS data. Our proposed mechanism may be tested by SAXS experiments on protein samples with mutated hydrophobic/charged residues.

## Electronic supplementary material

Supplementary material 1 (PDF 205 kb)
