# Supplementary material for: Determining structural ensembles of flexible multi-domain proteins using small-angle X-ray scattering and molecular dynamics simulations
Source: Protein Cell. 2015 May 6;6(8):619–23. doi: 10.1007/s13238-015-0162-4 (PMC4506289; doi:10.1007/s13238-015-0162-4)
Supplement: Supplementary file 1 — Supplementary material 1 (PDF 205 kb) [file 13238_2015_162_MOESM1_ESM.pdf]

Supplemental Materials

Determining Structural Ensembles of Flexible Multi-domain Proteins using Small-angle X-ray Scattering and Molecular Dynamics Simulations

Running title: **Investigating Flexible Multi-domain Proteins by SAXS and MD**

Key words: multi-domain proteins, protein flexibility, small-angle X-ray scattering, molecular dynamics simulations

Yonghui Zhang[1], Bin Wen[1], Junhui Peng[1], Xiaobing Zuo[2], Qingguo Gong[1], Zhiyong Zhang[1,*]

[1]Hefei National Laboratory for Physical Science at Microscale and School of Life Sciences, University of Science and Technology of China, Hefei, Anhui 230026, People's Republic of China; [2]Advanced Photon Source, Argonne National Laboratory, Chicago, IL 60437

*Corresponding author: Zhiyong Zhang, Tel: +86-551-63600854; Fax: +86-551-63600374; Email: zzyzhang@ustc.edu.cn

Table S1 Multiple MD simulations of FBP21-WWs

	**single-2μs**	**comb1-2μs**	**comb2-2μs**
Model 1[a]	2 us[b]	100 ns[c]	100 ns[d]
Model 2		100 ns	100 ns
Model 3		100 ns	100 ns
Model 4		100 ns	100 ns
Model 5		100 ns	100 ns
Model 6		100 ns	100 ns
Model 7		100 ns	100 ns
Model 8		100 ns	100 ns
Model 9		100 ns	100 ns
Model 10		100 ns	100 ns
Model 11		100 ns	100 ns
Model 12		100 ns	100 ns
Model 13		100 ns	100 ns
Model 14		100 ns	100 ns
Model 15		100 ns	100 ns
Model 16		100 ns	100 ns
Model 17		100 ns	100 ns
Model 18		100 ns	100 ns
Model 19		100 ns	100 ns
Model 20		100 ns	100 ns
**Total**	**2 us**	**2 us**	**2 us**

[a] Initial structure of the MD simulation taken from the NMR ensemble;
[b] Simulation time for each set of MD run;
[c&d] The two 100 ns MD simulations of the same model were run independently starting from different initial atomic velocities.

Figure S1 Domain motions of FBP21-WWs measured by angle values. (A) Positions of the three Cα atoms used to define the angle between domains. These Cα atoms (show in black beads) belong to Y16, D21 and Y60, respectively, which are all located in β sheets of the two WW domains. (B) The time evolution of the angle during the single-2μs MD simulation.

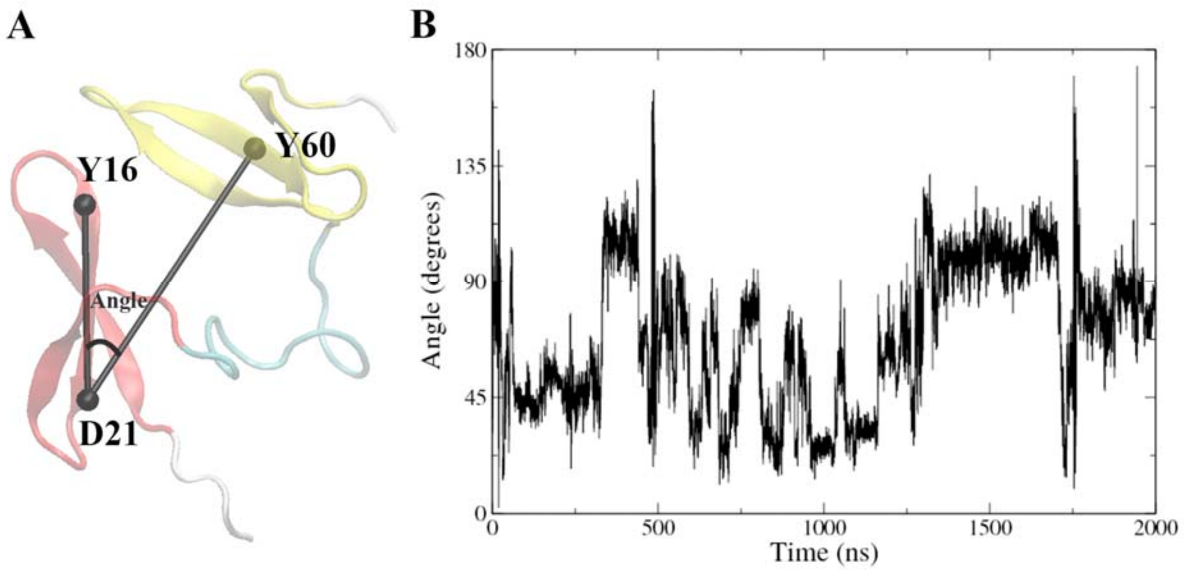

Materials and Methods

Protein cloning, expression and purification

The gene encoding the tandem WW domains of human FBP21 (residues 122-196) was amplified by PCR from the human brain cDNA library, and cloned into the *Nde*I/*Xho*I sites of plasmid pET-22b(+) (Novagen). The sequence of the DNA construct was verified by DNA sequencing. The protein was produced in *Escherichia coli* BL21 DE3 (Novagen). The bacteria were cultured in Luria–Bertani medium at 37°C to an OD_{600} of 0.6-0.8 and the expression of the protein was induced by addition of 0.1 mM isopropyl β-D-1-thiogalactopyranoside (IPTG) for 4 hours. The His-tagged protein was firstly extracted with a nickel-chelating column (Qiagen), and then purified using size-exclusion chromatography on a HiLoad 16/60 Superdex 75 column (GE Healthcare). For SAXS experiments, the purified protein was dialyzed into 20 mM Tris, 300 mM NaCl, 1 mM EDTA, pH 8.0.

SAXS data

The SAXS data of FBP21-WWs was collected at the beamline 12ID-B of the Advanced Photon Sources (APS) at Argonne National Laboratory, with a X-ray wavelength of 1.033 Å. Data were acquired from three concentrations (1.0, 3.0, and 5.0 mg/ml), which were then processed by the ATSAS package (Konarev et al., 2006; Petoukhov et al., 2012). After subtracting buffer scattering, the data curves from different concentrations were scaled and merged using PRIMUS (Konarev et al., 2003). The R_g of the protein was derived by Guinier analysis using AutoRg. GNOM (Semenyuk and Svergun, 1991) was employed for calculating the PDDF.

MD simulations

Each MD simulation was performed using the GROMACS-4.5.5 package (Hess et al., 2008) and the CHARMM27 force field (MacKerell et al., 1998). The protein was firstly put in a rhombic dodecahedron box with periodic boundary condition. The minimum distance between the solute and the box boundary was 1.2 nm. The box was then filled with TIP3P water molecules (Jorgensen et al., 1983). The system was energy-minimized by the steepest descent method, until the maximum force on any atom was smaller than 1000 kJ $mol^{-1}nm^{-1}$. After adding seven Na^+ ions to neutralize the charges on the protein, the system was energy-minimized again using the steepest descent and then the conjugate gradient method, until the maximum force on any atom was smaller than 50 kJ mol^{-1} nm^{-1}. The simulation was conducted by using the Verlet integration scheme with a 2 fs time step (Hockney et al., 1974). Before the production run, a 100 ps equilibration simulation with positional restraint was performed, using a force constant of 1000 kJ mol^{-1} nm^{-2}. The initial atomic velocities were generated according to a Maxwell distribution at 310 K. The simulation was performed under the constant NPT condition. The protein, solvent and ions were coupled separately to a temperature bath of 310K using the velocity rescaling algorithm (Bussi et al., 2007), with a relaxation time of 0.1 ps. The pressure was kept to 1 bar with a relaxation time of 0.5 ps and the compressibility of 4.5×10^{-5} bar^{-1} (Berendsen et al., 1984). The P-LINCS algorithm (Hess, 2008) was used to constrain all covalent bonds. The twin-range cutoff distances for the van der Waals interactions were set to be 0.9 and 1.4 nm, respectively, and the neighbor list was updated every 10 fs. The long-range electrostatic interactions were treated by the PME algorithm, with a tolerance of 10^{-5} and an interpolation order of 4 (Essmann et al., 1995).

The MD trajectories were analyzed by these tools in the GROMACS package. All the structures were created by VMD (Humphrey et al., 1996).

Principal component analysis

PCA on the 6 μs trajectory of FBP21-WWs consisted of following steps. (1) Snapshots were taken from the trajectory every 200 *ps*, so 30,000 conformations in total were used for PCA. Only 54 C_α atoms in the two WW domains were considered. (2) All the conformations were superimposed to the starting structure of single-2μs by the WW1 domain. (3) A 162×162 covariance matrix of atomic positional fluctuation, with each element as $\langle (x_i - \langle x_i \rangle)(x_j - \langle x_j \rangle) \rangle$, was constructed, where x_i is the Cartesian coordinate of atom *i*, and $\langle x_i \rangle$ means the ensemble average. (4) The covariance matrix was diagonalized to yield eigenvectors (PCA modes), and corresponding eigenvalues. (5) All the PCA modes were sorted in a decreasing order of their eigenvalues. Typically only a small number of PCA modes with the largest eigenvalues are dominant in collective motions of the protein (Berendsen and Hayward, 2000; Kitao and Go, 1999). These modes are also termed essential modes, and the subspace spanned by the essential modes is called essential subspace (Amadei et al., 1993).

Ensemble optimization method

EOM (Bernadó et al., 2007) was used to search an ensemble of structures from a trajectory, to best reproduce the experimental SAXS curve. The target function in EOM is

$$\chi = \left\{ \frac{1}{K-1} \sum_{m=1}^{K} \left[\frac{\mu I(q_m) - I_{exp}(q_m)}{\sigma(q_m)} \right]^2 \right\}^{1/2}, \quad \text{(S1)}$$

where K is the number of data points, and $q = 4\pi \sin\theta / \lambda$ is the momentum transfer where

2θ is the scattering angle and λ is the wavelength. $\sigma(q)$ are standard deviations, and μ is a scaling factor. $I(q)$ is the average of SAXS profiles from the ensemble

$$I(q)=\frac{1}{N}\sum_{n=1}^{N}I_n(q), \tag{S2}$$

where N is the number of conformations in the ensemble that would be much smaller than those in the trajectory. $I_n(q)$ is the theoretical SAXS profile of a single structure n calculated by CRYSOL (Svergun et al., 1995). In EOM, χ (Eqn. 3) is minimized by using the genetic algorithm, and then the optimal ensemble is picked.

References

Amadei, A., Linnsen, A.B.M., and Berendsen, H.J.C. (1993). Essential dynamics of proteins. Proteins 17, 412-425.

Berendsen, H.J.C., and Hayward, S. (2000). Collective protein dynamics in relation to function. Curr Opin Struct Biol 10, 165-169.

Berendsen, H.J.C., Postma, J.P.M., Vangunsteren, W.F., Dinola, A., and Haak, J.R. (1984). Molecular dynamics with coupling to an external bath. J Chem Phys 81, 3684-3690.

Bernadó, P., Mylonas, E., Petoukhov, M.V., Blackledge, M., and Svergun, D.I. (2007). Structural characterization of flexible proteins using small-angle X-ray scattering. J Am Chem Soc 129, 5656-5664.

Bussi, G., Donadio, D., and Parrinello, M. (2007). Canonical sampling through velocity rescaling. J Chem Phys 126, 014101.

Essmann, U., Perera, L., Berkowitz, M.L., Darden, T., Lee, H., and Pedersen, L.G. (1995). A Smooth Particle Mesh Ewald Method. J Chem Phys 103, 8577-8593.

Hess, B. (2008). P-LINCS: A Parallel Linear Constraint Solver for Molecular Simulation. J

Chem Theory Comput 4, 116-122.

Hess, B., Kutzner, C., van der Spoel, D., and Lindahl, E. (2008). GROMACS 4: algorithms for highly efficient, load-balanced, and scalable molecular simulation. J Chem Theory Comput 4, 435-447.

Hockney, R.W., Goel, S.P., and Eastwood, J.W. (1974). Quiet High-Resolution Computer Models of a Plasma. J Comput Phys 14, 148-158.

Humphrey, W., Dalke, A., and Schulten, K. (1996). VMD: visual molecular dynamics. J Mol Graphics 14, 33-38.

Jorgensen, W.L., Chandrasekhar, J., Madura, J.D., Impey, R.W., and Klein, M.L. (1983). Comparison of Simple Potential Functions for Simulating Liquid Water. J Chem Phys 79, 926-935.

Kitao, A., and Go, N. (1999). Investigating protein dynamics in collective coordinate space. Curr Opin Struct Biol 9, 164-169.

Konarev, P.V., Petoukhov, M.V., Volkov, V.V., and Svergun, D.I. (2006). ATSAS 2.1, a program package for small-angle scattering data analysis. J Appl Cryst 39, 277-286.

Konarev, P.V., Volkov, V.V., Sokolova, A.V., Koch, M.H.J., and Svergun, D.I. (2003). PRIMUS: a Windows PC-based system for small-angle scattering data analysis. J Appl Cryst 36, 1277-1282.

MacKerell, A.D., Bashford, D., Bellott, M., Dunbrack, R.L., Evanseck, J.D., Field, M.J., Fischer, S., Gao, J., Guo, H., Ha, S., *et al.* (1998). All-atom empirical potential for molecular modeling and dynamics studies of proteins. J Phys Chem B 102, 3586-3616.

Petoukhov, M.V., Franke, D., Shkumatov, A.V., Tria, G., Kikhney, A.G., Gajda, M., Gorba, C.,

Mertens, H.D.T., Konarev, P.V., and Svergun, D.I. (2012). New developments in the ATSAS program package for small-angle scattering data analysis. J Appl Cryst 45, 342-350.

Semenyuk, A.V., and Svergun, D.I. (1991). GNOM - a program package for small-angle scattering data-processing. J Appl Cryst 24, 537-540.

Svergun, D., Barberato, C., and Koch, M.H.J. (1995). CRYSOL - a program to evaluate x-ray solution scattering of biological macromolecules from atomic coordinates. J Appl Crystallogr 28, 768-773.
